# Factor Analysis Related to the Change in Activities of Daily Living Performance of Stroke Patients

**DOI:** 10.1155/2023/6147413

**Published:** 2023-12-23

**Authors:** Hye Eun Kim, Ki Hun Cho

**Affiliations:** Department of Physical Therapy, Korea National University of Transportation, 61 Daehak-ro, Jeungpyeong-gun, Chungbuk 27909, Republic of Korea

## Abstract

This study is aimed at investigating the factors influencing the change (improvement) in the performance of activities of daily living (ADL) in stroke patients. This study utilized data collected from 123 stroke patients at admission and discharge from a local rehabilitation hospital in South Korea between 2019 and 2022. We analyzed the correlations between ADL (Korean version of the Modified Barthel Index) and spasticity (Modified Ashworth Scale), muscle strength (Manual Muscle Test), gait ability (Functional Ambulation Category), cognitive function (Korean version of Mini Mental Status Examination), upper extremity function (Manual Function Test), and balance function (Berg Balance Scale). In addition, to identify the factors influencing the change in ADL in line with the 3 months of rehabilitation, a multiple regression analysis was performed. The results of the correlation analysis showed that balance function (*r* = 0.925, *p* < 0.01) and gait ability (*r* = 0.921, *p* < 0.01) had strong positive correlations with ADL. Multiple regression analysis (*F* = 34.547, *p* ≤ 0.001, adj.*R*^2^ = 0.466) indicated that changes in the upper extremity (*β* = 0.494, *p* ≤ 0.001), cognitive (*β* = 0.197, *p* = 0.008), and balance (*β* = 0.163, *p* = 0.029) functions influenced changes in ADL. Our results found that the recovery of upper extremity function along with the improvement of cognitive and balance functions had a significant effect on the improvement of independence in ADL in stroke patients.

## 1. Introduction

In South Korea, stroke is the 4th leading cause of death, with 607,862 patients visiting hospitals due to stroke in 2020, an increase of approximately 6% from 573,379 in 2016. In particular, as the trend continues, the risk increases [1]. Poststroke motor recovery is influenced by various factors, including lesion location, extent of damage, age, etiology, and medical history; however, predicting the level and extent of poststroke recovery remains difficult [2]. Therefore, accurately assessing functional movement and performance of activities of daily living (ADL) in stroke patients is important to achieve the goal of functional recovery for independent daily living through rehabilitation [3].

ADL in stroke patients are reportedly influenced by cognitive, upper extremity, lower extremity, gait, and balance functions [4–6]. Regarding daily activities, cognitive impairment is one of the main causes of dependency in stroke patients for daily activities and is a critical predictor and determinant of the level of functional recovery [7, 8]. Upper extremity function is an essential factor in daily living, and activities such as self-care, personal hygiene, feeding, and dressing are highly dependent on upper extremity function [9–11]. Additionally, lower extremity dysfunction causes difficulties in transfer and ambulation, preventing independent daily living [12]. Furthermore, lower extremity dysfunction can impair balance function, which is strongly correlated with gait ability, resulting in an asymmetric shift in weight and reduced postural control, ultimately leading to dependent daily activities [13].

Recently, in South Korea, numerous studies have investigated the factors that influence the performance of ADL in stroke patients [14–16]. Kim reported that the type of hemiplegia, cognitive function, and depression were factors influencing the performance of ADL in stroke patients [15]. Lee performed a multiple regression analysis to predict the performance of ADL in stroke patients using data collected at admission. This study reported that gait and cognitive functions had a strong relative influence on the performance of daily activities. However, previous studies have simply analyzed the factors influencing ADL (at the time of admission or discharge) without identifying the factors that influence the change (improvement) in ADL performance through rehabilitation [16].

Analyzing the factors that influence improvement in the performance of ADL after stroke and incorporating the identified influencing factors into the treatment process is important for promoting functional recovery [17]. In addition, by identifying factors that improve the performance of ADL, more accurate treatment-related information can be provided to patients and their families. Furthermore, detailed treatment goals can be established to increase therapeutic motivation [18]. Thus, this study utilized data collected from stroke patients at admission and discharge from a local rehabilitation hospital in South Korea to analyze the factors influencing the change (improvement) in the performance of ADL.

## 2. Subjects and Methods

### 2.1. Participants and Procedures

The sample size was estimated using G^∗^Power version 3.1.9.7. The required number of participants was *n* = 123 (testing power, 0.90; significance level, *α* = 0.05; effect size, 0.15; and independent variables, 6). This retrospective study analyzed the medical records of 123 patients admitted to a local rehabilitation hospital for stroke (between January 2019 and December 2022). The inclusion criteria were a diagnosis of stroke based on magnetic resonance imaging findings, no psychiatric history before the stroke, and discharge after 3 months of rehabilitation training. Individuals with neurological disorders other than stroke and those with lesions in the cerebellum, brain stem, or cerebral hemisphere on either side were excluded. This study was approved by the Korea National University of Transportation Institutional Review Board (KNUT IRB-2023-05) and conducted per the approved guidelines. All patients provided informed consent in accordance with the Declaration of Helsinki before commencing the study.

The data collected for the general characteristics included sex, age, poststroke duration, etiology, and paretic side. The data collected for the medical characteristics included spasticity (Modified Ashworth Scale, MAS), muscle strength (Manual Muscle Test, MMT) of the paretic side's upper and lower extremities, gait ability (Functional Ambulation Category, FAC), cognitive function (Korean version of Mini Mental Status Examination, K-MMSE), upper extremity function (Manual Function Test, MFT), balance function (Berg Balance Scale, BBS), and ADL performance (Korean version of the Modified Barthel Index, K-MBI). The general and medical characteristics of the participants are presented in Table 1. The general and medical characteristics were collected using a computerized questionnaire administered at the hospital. Data pertaining to general characteristics were based on the medical record documentation by the ward nurses. Medical characteristics, cognitive function, upper extremity function, and ADL performance were recorded based on assessments by occupational therapists. Physical therapists recorded spasticity, muscle strength, gait ability, and balance. Medical characteristics included data collected at admission and discharge after 3 months of rehabilitation training to examine the changes resulting from rehabilitation.

Correlation analysis was used to confirm the correlation between the K-MBI and independent variables. In addition, to identify the factors influencing changes in ADL performance, a multiple regression analysis was performed. Personal information was anonymized, and all documents were coded for management purposes. All data were used solely for research purposes.

### 2.2. Outcome Measurements

#### 2.2.1. Spasticity of the Paretic Upper and Lower Extremities

The spasticity of the paretic upper and lower extremities was assessed using the MAS. The MAS is the most widely used tool to assess muscle stiffness in clinical practice and uses a 6-point scale [19]. The interrater reliability (*r*) for elbow and knee joint flexion was 0.67–0.73 and 0.45, respectively, and the test-retest reliability for elbow, wrist, and knee joint flexion was 0.77–0.94 [2].

#### 2.2.2. Muscle Strength of the Paretic Upper and Lower Extremities

The muscle strength of the paretic side upper and lower extremities was assessed using MMT. The MMT is a widely used tool to assess muscle strength based on manual resistance and gravity [20]. In this study, measurements were taken for the shoulder flexor, elbow extensor, hip joint extensor, knee joint extensor, and ankle dorsiflexor muscles on the paretic side.

#### 2.2.3. Gait Ability

Gait ability was assessed using the FAC. The FAC uses a 6-point scale (range, 0–5) [21] as follows: “The patient is a non-functional ambulator (0),” “The patient is an ambulator, requiring physical assistance of two or more people (1),” “The patient is an ambulator, requiring continuous assistance of one person upon motion or balance control (3),” “The patient is an ambulator without physical assistance but with linguistic supervision (4),” “The patient is an ambulator requiring assistance on stairs and irregular surfaces (5).”

#### 2.2.4. Cognitive Function

Cognitive function was assessed using the K-MMSE. The K-MMSE is a useful tool to quantitatively evaluate the level of cognitive decline [22], which consists of “time orientation (five items),” “space orientation (five items),” “memory (three items),” “attention and calculation (five items),” “recall (three items),” “language (eight items),” and “visuospatial construction (one item).” The test-retest reliability of the K-MMSE in older persons is 0.86 [23].

#### 2.2.5. Upper Extremity Function

Upper extremity function was assessed using the MFT. The MFT consists of eight items regarding upper extremity motion, grip strength, and finger dexterity, with a total score of 32 [24]. The interrater and test-retest reliability, both ≥0.95, are used for assessing the upper extremity functions [25].

#### 2.2.6. Balance Function

The balance function was assessed using the BBS. The BBS assesses balance function in older people and stroke patients in a standing position or during movement. The tool consists of 14 items (from the lowest score of 0 to the highest score of 4), for a total score of 56. The scores 0–20 indicate the use of a wheelchair; 21–40 indicate the need for an assistive tool or assistance; and ≥41 indicate independent gait [26]. The intrarater and interrater reliability were 0.98 and 0.97, respectively, confirming BBS as a highly reliable tool for assessing balance function [27].

#### 2.2.7. ADL Performance

ADL performance was assessed using the K-MBI. The K-MBI consists of 11 items (personal hygiene, bathing, having a meal, bladder control, bowel control, primary care, gait ability, climbing stairs, getting dressed, ambulation or wheelchair use, and chair/bed transfer) [28]. Each item is rated on a 5-point scale, with a total score of 100. Scores of 0–24 indicate complete dependency, 25–49 indicate maximal dependency, 50–74 indicate partial dependency, 75–90 indicate slight dependency, 91–99 indicate minimal dependency, and 100 indicate complete independency. The test-retest and interrater reliability are 0.89 and 0.95, respectively [29].

### 2.3. Statistical Analysis

SPSS (version 27.0; IBM Corp., Armonk, NY, USA) was used for statistical analyses. The general and medical characteristics of the participants were determined using descriptive statistics and frequency analyses. To determine the correlations between ADL performance at the time of admission and other medical characteristics, we analyzed Spearman's and Pearson's correlations between ADL performance and spasticity and muscle strength of the paretic upper and lower extremities, gait ability, cognitive function, upper extremity function, and balance function. In addition, to identify the factors influencing the change in ADL performance in line with 3 months of rehabilitation training, a multiple regression analysis was performed. The magnitude of the change in ADL performance served as the dependent variable, and the changes in other medical characteristics were used as independent variables. The significance level was set at *p* < 0.05.

## 3. Results

### 3.1. Correlation between K-MBI and MAS, MMT, FAC, and K-MMSE

Spearman's correlation analysis was used to determine correlations among spasticity (MAS), muscle strength (MMT), gait ability (FAC), and ADL performance (K-MBI) (Table 2).

ADL performance showed a very strong positive correlation with gait ability (*r* = 0.921, *p* < 0.01), a moderately strong positive correlation with muscle strength (*r* = 0.629–0.805, *p* < 0.01), and a poor negative correlation with spasticity (upper extremity) (*r* = −0.211, *p* < 0.05).

### 3.2. Correlation between K-MBI and K-MMSE, BBS, and MFT

Pearson's correlation analysis was used to determine the correlations among cognitive function (K-MMSE), balance function (BBS), upper extremity function (MFT), and ADL performance (K-MBI) (Table 3).

Activities of daily living performance showed a strong positive correlation with balance function (*r* = 0.925, *p* < 0.01), a strong positive correlation with cognitive function (*r* = 0.731, *p* < 0.01), and a moderate positive correlation with upper extremity function (*r* = 0.606, *p* < 0.01).

### 3.3. Factors Related to the Change in ADL Performance

To identify the factors that influenced the change in ADL performance, a multiple regression analysis was performed. The results indicated that the fit of the regression model was acceptable (*F* = 34.547, *p* ≤ 0.001), with an explanatory power of 46.6% (adj.*R*^2^ = 0.466). The null hypothesis was rejected, and the alternative hypothesis was accepted based on changes in cognitive (*β* = 0.197, *p* = 0.008), upper extremity (*β* = 0.494, *p* ≤ 0.001), and balance (*β* = 0.163, *p* = 0.029) functions. Thus, changes in cognitive, upper extremity, and balance function were identified as variables that significantly impacted the change in ADL performance. Furthermore, the B sign was positive (+), indicating that for every one-unit increase in cognitive function, the change in ADL performance increased by 0.442. Similarly, for every one-unit increase in upper extremity and balance function, the change in ADL performance increased by 0.998 and 0.246, respectively. The standardization coefficient *β* was used to compare changes in cognitive, upper extremity, and balance functions. The relative influence was strongest for changes in upper extremity function (*β* = 0.494), followed by cognitive function (*β* = 0.197) and balance function (*β* = 0.163) (Table 4).

## 4. Discussion

To analyze the factors that influence ADL performance in stroke patients, data from 123 stroke patients were collected from their medical records. Correlations of ADL performance with spasticity, muscle strength, gait ability, cognitive function, upper extremity function, and balance function were analyzed. Through multiple regression analysis, the factors influencing changes in ADL performance were identified.

As a result of the correlation analysis, balance function (BBS) and gait ability (FAC) were shown to have strong positive correlations with ADL performance. Most studies on stroke patients, in which a correlation analysis was performed on ADL performance, reported a strong correlation with balance function. Moon and Won analyzed the correlation between ADL performance and cognitive, manual, and balance functions in stroke patients and reported a strong positive correlation with balance function (*r* = 0.827, *p* < 0.05). Notably, balance function was correlated with all subcategories of ADL performance (personal hygiene, bathing, feeding, toilet, stair climbing, dressing, bowel control, bladder control, ambulation, and wheelchair or chair/bed transfer) [30]. Cho and Kim analyzed the correlation between ADL performance in stroke patients and balance and cognitive functions and reported that ADL performance was positively correlated with balance function (*r* = 0.580, *p* < 0.05) [31]. Jung et al. also reported a positive correlation (*r* = 0.529, *p* < 0.01) between ADL performance in stroke patients and balance function [32].

The gait ability of stroke patients is strongly correlated with their quality of life, making it the most significant factor in the rehabilitation of stroke patients [33]. Moreover, recovery of gait ability is considered a critical factor for the functional independence of stroke patients in their daily living performance [34]. An et al. conducted a correlation study on 40 stroke patients and showed that ADL performance was strongly correlated with mobility based on the BBS. Furthermore, mobility and gait ability were shown to be closely associated with independence in daily activities [35]. Lee et al. also analyzed the correlations among balance function, functional gait ability, visual perception, and ADL in stroke patients. They reported a close correlation between functional gait ability and ADL. Similarly, in this study, ADL performance showed a very strong positive correlation with balance function (*r* = 0.925, *p* < 0.01) and gait ability (*r* = 0.921, *p* < 0.01) [36]. While the results of this study are not novel findings and are in agreement with those of previous studies, they are likely to make a significant contribution to the field by highlighting the importance of assessing balance function and gait ability in evaluating ADL performance in stroke patients.

The most interesting findings of this study are from the factor analysis conducted to identify the factors influencing the change in ADL performance after 3 months of rehabilitation training. Using multiple regression analysis, we identified the factors influencing changes in ADL performance in the following order of strongest influence: changes in the upper extremity (*β* = 0.494), cognitive (*β* = 0.197), and balance (*β* = 0.163) functions. In the correlation analysis based on assessments at admission, ADL performance was strongly correlated with balance function, gait ability, and cognitive function. However, after 3 months of rehabilitation training, changes in upper extremity function were shown to have the strongest impact on changes in ADL performance.

Improvement in upper extremity function in stroke patients is essential for returning home and to the community [37]. Additionally, the level of poststroke upper extremity function is a critical factor in predicting prognosis [38]. From a cerebrovascular pathological perspective, injuries to the middle cerebral artery are more common than those to the anterior cerebral artery. Moreover, this area is more likely to be damaged than others because of the wide distribution of cerebral motor neurons that govern the upper extremity [38]. In clinical practice, the importance of recovering upper extremity function has been recognized, and various therapeutic approaches have been applied in poststroke rehabilitation. Nevertheless, the high probability of damage, slow recovery, and difficulties in brain reorganization (difficulties in repeated motor learning in the upper extremities compared to the lower extremities) still pose challenges in rehabilitation aimed at recovering upper extremity function [39]. Moreover, the treatments provided by physical therapists in South Korea tend to prioritize improvements in balance function and gait ability over the recovery of upper extremity function. However, various studies have reported that the recovery of upper extremity function can enhance functional movements [37, 40, 41]. A study by Bang and Cho, in which stroke patients underwent arm training, reported that improvements in balance function and gait ability followed improvements in upper extremity function [40]. Oh and Kim reported that upper limb robotic therapy combined with conventional rehabilitation therapy could enhance upper extremity function, ADL, and cognitive function in stroke patients [41]. The findings of previous studies and this study suggest that the recovery of upper extremity function, in addition to improvements in cognitive and balance functions, has an important effect on the enhanced independence of stroke patients in ADL performance. Therefore, in clinical practice, therapeutic approaches to improve cognitive and balance functions should continue to be recommended, but they should also include various and consistent therapeutic approaches to improve upper extremity function.

### 4.1. Limitations

This study has some limitations. This was a retrospective study, and potential confounders could not be completely controlled. In particular, a previous study reported that participants with poststroke depression had reduced independence in ADL performance [42], but in this regression analysis, the subjects' depression was not included as an analysis factor. In future studies, psychological factors such as depression and anxiety should be included as analysis factors. In addition, as the analyzed data were from stroke patients admitted to a single local rehabilitation hospital in South Korea during a set period, it is difficult to generalize the results to all stroke patients. Although the assessments of clinical characteristics in this study were performed by licensed physical and occupational therapists, variations in accuracy caused by differences in clinical career length (range, 1–15 years) could not be completely controlled. Future, long-term retrospective studies with larger sample sizes are warranted.

## 5. Conclusion

This study utilized data collected from stroke patients at admission and discharge from a local rehabilitation hospital in South Korea to analyze the factors influencing the change (improvement) in ADL performance. We found that the recovery of upper extremity function, along with the improvement of cognitive and balance functions, had a significant effect on the improvement of independence in performing ADL in stroke patients. We think that the results of this study may help predict the prognosis of stroke patients and establish basic data for therapeutic interventions.

## Figures and Tables

**Table 1:** General and medical characteristics of the participants (*N* = 123).

Parameters		Admission			Discharge			△ values
		*N*	%	Mean ± SD	*N*	%	Mean ± SD	Mean ± SD
Gender	M/F	67/56	54.5/45.5					
Stroke type	I/H	88/35	71.5/28.5					
Hemiplegic side	R/L	68/55	55.3/44.7					
Age	Years			65.37 ± 13.37				
Onset duration	Days			46.62 ± 52.45				

MAS								
Upper extremity	0	103	83.7		104	84.6		
	1	15	12.2		14	11.4		
	1+	3	2.4		3	2.4		
	2	1	1.6		1	1.6		
Lower extremity	0	109	88.6		110	89.4		
	1	13	10.6		12	9.8		
	2	1	0.8		1	0.8		

MMT								
Shoulder flexor	Zero	5	4.1		2	1.6		
	Trace	10	8.1		9	7.3		
	Poor	33	26.8		24	19.5		
	Fair	48	39.0		56	45.5		
	Good	27	22.0		32	26.0		
Elbow extensor	Zero	6	4.9		3	2.4		
	Trace	20	16.3		13	10.6		
	Poor	27	22.0		25	20.3		
	Fair	47	38.2		50	40.7		
	Good	23	18.7		32	26.0		
Hip extensor	Zero	3	2.4		1	0.8		
	Trace	10	8.1		5	4.1		
	Poor	20	16.3		15	12.2		
	Fair	47	38.2		44	35.8		
	Good	43	35.0		58	47.2		
Knee extensor	Zero	3	2.4		1	0.8		
	Trace	9	7.3		5	4.1		
	Poor	17	13.8		15	12.2		
	Fair	49	39.8		44	35.8		
	Good	45	36.6		58	47.2		
Ankle dorsiflexor	Zero	6	4.9		4	3.3		
	Trace	14	11.4		12	9.8		
	Poor	25	20.3		16	13.0		
	Fair	48	39.0		56	45.5		
	Good	30	24.4		35	28.5		

K-MMSE, score				22.22 ± 8.11			24.53 ± 6.54	2.31 ± 3.18

MFT, score				16.49 ± 10.16			18.07 ± 9.70	1.58 ± 3.53

BBS, score				30.86 ± 19.22			33.83 ± 18.13	2.97 ± 4.74

K-MBI, score								
Personal hygiene				3.11 ± 1.54			3.56 ± 1.23	0.46 ± 0.76
Bathing self				2.73 ± 1.70			2.98 ± 1.64	0.24 ± 0.59
Feeding				6.27 ± 2.67			6.98 ± 2.35	0.72 ± 1.30
Toilet				5.68 ± 3.57			6.51 ± 3.29	0.83 ± 1.40
Stair climbing				3.11 ± 3.60			3.54 ± 3.76	0.42 ± 1.00
Dressing				5.72 ± 3.16			6.29 ± 2.87	0.58 ± 1.30
Bowel control				7.74 ± 3.31			8.29 ± 2.76	0.55 ± 1.71
Bladder control				7.68 ± 3.30			8.23 ± 2.83	0.54 ± 1.63
Ambulation or wheelchair				5.98 ± 5.00			6.76 ± 4.88	0.79 ± 1.50
Chair/bed transfer				8.23 ± 5.10			9.20 ± 4.64	0.97 ± 1.91
Total				56.24 ± 29.44			62.34 ± 26.75	6.10 ± 7.12

M: male; F: female; I: infarction; H: hemorrhage; R: right; L: left; MAS: Modified Ashworth Scale; MMT: Manual Muscle Test; FAC: Functional Ambulation Categories; K-MMSE: Korean version of Mini Mental Status Examination; MFT: Manual Function Test; BBS: Berg Balance Scale; K-MBI: Korean version of Modified Barthel Index.

**Table 2:** Correlation between activities of daily living performance and MAS, MMT, and FAC (*N* = 123).

Parameters	K-MBI	MAS U/E	MAS L/E	MMT SF	MMT EE	MMT HE	MMT KE	MMT ADF
MAS U/E	-0.211^∗^							
MAS L/E	-0.155	1.000						
MMT SF	0.662^∗∗^	-0.389^∗∗^	-0.268^∗∗^					
MMT EE	0.629^∗∗^	-0.437^∗∗^	-0.321^∗∗^	0.888^∗∗^				
MMT HE	0.805^∗∗^	-0.365^∗∗^	-0.291^∗∗^	0.664^∗∗^	0.645^∗∗^			
MMT KE	0.793^∗∗^	-0.342^∗∗^	-0.255^∗∗^	0.660^∗∗^	0.652^∗∗^	0.918^∗∗^		
MMT ADF	0.742^∗∗^	-0.359^∗∗^	-0.237^∗∗^	0.666^∗∗^	0.665^∗∗^	0.833^∗∗^	0.849^∗∗^	
FAC	0.921^∗∗^	-0.253^∗∗^	-0.137^∗∗^	0.639^∗∗^	0.640^∗∗^	0.826^∗∗^	0.833^∗∗^	0.812^∗∗^

^∗^ *p* < 0.05, ^∗∗^*p* < 0.01. MAS: Modified Ashworth Scale; MMT: Manual Muscle Test; FAC: Functional Ambulation Categories; K-MBI: Korean version of Modified Barthel Index; U/E: upper extremity; L/E: lower extremity, SF: shoulder flexor; EE: elbow extensor; HE: hip extensor; KE: knee extensor; ADF: ankle dorsiflexor.

**Table 3:** Correlation between activities of daily living performance and K-MMSE, BBS, and MFT (*N* = 123).

Parameters	K-MBI	MMSE	BBS
K-MMSE	0.731^∗∗^		
BBS	0.925^∗∗^	0.689^∗∗^	
MFT	0.606^∗∗^	0.362^∗∗^	0.639^∗∗^

^∗∗^ *p* < 0.01. K-MMSE: Korean version of Mini Mental Status Examination; MFT: Manual Function Test; BBS: Berg Balance Scale; K-MBI: Korean version of Modified Barthel Index.

**Table 4:** Factors influencing change of activities of daily living performance (*N* = 123).

Parameters	Discharge-admission				
	*B*	SE	*β*	*t* (*p*)	VIF
Constant	2.774	0.624		4.442	
△K-MMSE	0.442	0.165	0.197	2.683 (.008)	1.204
△MFT	0.998	0.154	0.494	6.471 (.000)	1.297
△BBS	0.246	0.111	0.163	2.213 (.029)	1.215
*R* & adj.*R*^2^	0.682 & 0.466				
*F* (*p*)	34.547 (0.000)				
Durbin-Watson	2.079				

B: beta input value; SE: standard error; VIF: variance inflation factor; K-MMSE: Korean version of Mini Mental Status Examination; MFT: Manual Function Test; BBS: Berg Balance Scale; K-MBI: Korean version of Modified Barthel Index.

## Data Availability

The data sets used and analyzed during the current study are available from the corresponding author on reasonable request.
